# P-1472. Cefiderocol Remains Highly Active Against Carbapenemase-Producing Enterobacterales

**DOI:** 10.1093/ofid/ofae631.1642

**Published:** 2025-01-29

**Authors:** Boudewijn L DeJonge, Sean T Nguyen, Jason J Bryowsky, Joshua Maher, Rodrigo Mendes, Christopher M Longshaw, Miki Takemura, Yoshinori Yamano

**Affiliations:** Shionogi Inc., Florham Park, New Jersey; Shionogi Inc., Florham Park, New Jersey; Shionogi Inc., Florham Park, New Jersey; JMI Laboratories, North Liberty, Iowa; JMI Labs, Liberty City, Iowa; Shionogi B.V., London, England, United Kingdom; Shionogi & Co., Ltd, Toyonaka, Osaka, Japan; Shionogi & Co., Ltd., Toyonaka, Osaka, Japan

## Abstract

**Background:**

Carbapenem-resistance in Enterobacterales is mediated by the acquisition of β-lactamases, including serine carbapenemases (KPC and OXA-48) and metallo-β-lactamases (NDM, IMP and VIM). Cefiderocol (CFDC) is a siderophore conjugated cephalosporin with good stability against all classes of β-lactamases. In this study, *in vitro* activity for CFDC and comparator agents was assessed against meropenem and/or imipenem non-susceptible carbapenemase-producing (CP) isolates that were collected between 2020-2022 in Europe and the USA as part of the SENTRY Antimicrobial Surveillance Program.

**Table 1.**
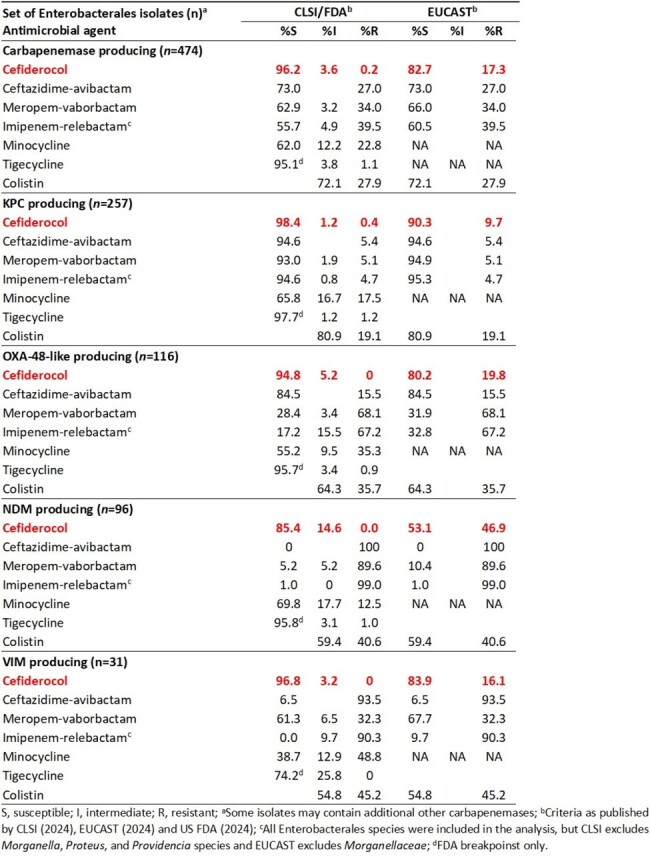
Susceptibility for cefiderocol and comparator agents against meropenem and/or imipenem non-susceptible carbapenemase-producing Enterobacterales isolates collected from European and US hospitals as part of the SENTRY surveillance program during 2020-2022.

**Methods:**

A total of 24,084 Enterobacterales were collected and minimum inhibitory concentrations (MIC) for compactor agents were determined according to CLSI guidelines using broth microdilution with cation-adjusted Mueller-Hinton broth (CAMHB), while for CFDC iron-depleted CAMHB was used. Susceptibility was assessed according to 2024 CLSI, FDA, and EUCAST breakpoints. Isolates non-susceptible for meropenem and/or imipenem, based on CLSI breakpoints (*n*=2,029), were subjected to whole genome sequencing to determine their carbapenemase content.

**Results:**

474 isolates (23.4%) produced carbapenemases, most of them being KPC enzymes (54.2%), followed by OXA-48-like (24.5%), NDM (20.3%), and VIM (6.5%). Three isolates (0.6%, all *S. marcescens*) produced SEM enzymes and one *K. pneumoniae* (0.2%) produced an IMP. CFDC was highly active against CP isolates, with >90% of the KPC-, OXA-48-like, VIM-, IMP-, and SEM-producing and 85.4% of NDM-producing isolates being susceptible according to FDA/CLSI breakpoints (Table 1). While susceptibilities were lower when applying EUCAST breakpoints, because of the number of isolates with CFDC MIC values of 4 µg/mL, CFDC remained one of the most active agents. Compared to β-lactam-β-lactamase inhibitor combinations, CFDC showed higher susceptibility against the CP isolates, mainly the result of the increased stability of CFDC against metallo-β-lactamases.

**Conclusion:**

CFDC is active against CP Enterobacterales, regardless of the carbapenemase produced. In contrast, β-lactam-β-lactamase inhibitor combinations showed variable activity. CFDC should be considered as a treatment option when CP Enterobacterales are encountered.

**Disclosures:**

**Boudewijn L. DeJonge, PhD**, Shionogi Inc.: Employee **Sean T. Nguyen, PharmD**, Shionogi Inc.: Employee **Jason J. Bryowsky, PharmD, MS**, Shionogi: Employee **Rodrigo Mendes, PhD**, Shionogi & Co., Ltd.: Grant/Research Support **Christopher M. Longshaw, PhD**, Shionogi BV: Employee **Miki Takemura, n/a**, Shionogi & Co., Ltd.: Employee **Yoshinori Yamano, PhD**, Shionogi & Co., Ltd.: Employee

